# A CRISPR‐nonhomologous end‐joining‐based strategy for rapid and efficient gene disruption in *Mycobacterium abscessus*

**DOI:** 10.1002/mlf2.70007

**Published:** 2025-04-23

**Authors:** Sanshan Zeng, Yanan Ju, Md Shah Alam, Ziwen Lu, H. M. Adnan Hameed, Lijie Li, Xirong Tian, Cuiting Fang, Xiange Fang, Jie Ding, Xinyue Wang, Jinxing Hu, Shuai Wang, Tianyu Zhang

**Affiliations:** ^1^ State Key Laboratory of Respiratory Disease, Guangzhou Institutes of Biomedicine and Health Chinese Academy of Sciences Guangzhou China; ^2^ Guangdong‐Hong Kong‐Macao Joint Laboratory of Respiratory Infectious Diseases, Guangzhou Institutes of Biomedicine and Health Chinese Academy of Sciences Guangzhou China; ^3^ China‐New Zealand Joint Laboratory on Biomedicine and Health, Guangzhou Institutes of Biomedicine and Health Chinese Academy of Sciences Guangzhou China; ^4^ University of Chinese Academy of Sciences Beijing China; ^5^ School of Basic Medical Sciences, Division of Life Science and Medicine University of Science and Technology of China Hefei China; ^6^ State Key Laboratory of Respiratory Disease Guangzhou Chest Hospital Guangzhou China; ^7^ Institute of Physical Science and Information Technology Anhui University Hefei China; ^8^ Guangzhou National Laboratory Guangzhou China

**Keywords:** CRISPR‐Cas12a, DSB repair, *Mycobacterium abscessus*, NHEJ, NrgA

## Abstract

*Mycobacterium abscessus*, a fast‐growing, non‐tuberculous mycobacterium resistant to most antimicrobial drugs, causes a wide range of serious infections in humans, posing a significant public health challenge. The development of effective genetic manipulation tools for *M. abscessus* is still in progress, limiting both research and therapeutic advancements. However, the clustered regularly interspaced short palindromic repeats (CRISPR)‐associated protein (Cas) systems have emerged as promising tools for generating highly specific double‐strand breaks (DSBs) in its genome. One of the mechanisms that repair these DSBs is the error‐prone nonhomologous end‐joining (NHEJ) pathway, which facilitates targeted gene editing. In this study, we introduced a novel application of the CRISPR‐NHEJ approach in *M. abscessus*. We demonstrated that NrgA from *M. marinum* plays a crucial role in repairing DSBs induced by the CRISPR‐Cas system in *M. abscessus*. Contrary to previous findings, our study also revealed that inhibiting or overexpressing components of homologous recombination/single‐strand annealing significantly reduces the efficiency of NHEJ repair in *M. abscessus*. This discovery challenges current perspectives and suggests that NHEJ repair in *M. abscessus* may involve components from both homologous recombination and single‐strand annealing pathways, highlighting the complex interactions among the three DSB repair mechanisms in *M. abscessus*.

## Impact statement

Antibiotic resistance has turned *Mycobacterium abscessus* into a growing public health threat, making it urgent to find new treatment strategies. Here, we present a groundbreaking application of the CRISPR‐based nonhomologous end‐joining gene editing technique, providing powerful new tools to study the genetics of *M. abscessus*. We also identified the crucial role of NrgA from *M. marinum* in DNA repair and revealed complex interactions among various DNA repair pathways. These findings not only deepen our understanding of mycobacterial genetics but also provide new possibilities for developing targeted therapies to combat this highly drug‐resistant pathogen.

## INTRODUCTION

The prevalence of lung infections caused by non‐tuberculous mycobacteria (NTM) has been rising annually, posing an escalating threat to public health. Among these NTM, *Mycobacterium abscessus* is one of the primary causative agents in both the United States and the humid regions of southern coastal China^1^, ^2^, ^3^. Besides pulmonary infections, *M. abscessus* can infect the skin, soft tissues, bone, and various other parts of the human body^4^. The intrinsic resistance of *M. abscessus* to a broad spectrum of antibiotics not only complicates the treatment but also requires the use of multidrug regimens and prolonged therapies^5^. The threat posed by *M. abscessus* underscores the urgent need for the development of genetic tools aimed at drug target identification, elucidating resistance mechanisms, and advancing vaccine research.

The Clustered Regularly Interspaced Short Palindromic Repeats (CRISPR)‐associated protein (Cas) systems have been widely applied as efficient gene editing tools in various organisms^6^. Among these systems, CRISPR‐associated protein 9 (Cas9) and Cas12a (formerly known as Cpf1) are the most commonly used and important nucleases^7^, ^8^. Guided by the corresponding CRISPR‐RNA, Cas9 and Cpf1 can precisely recognize and bind to the target DNA sequence. This process relies on the complementary base pairing between the CRISPR‐RNA and the target DNA sequence. Once matched, the CRISPR effector nuclease induces double‐strand breaks (DSBs) at designated loci within the target DNA. However, DSBs can be highly detrimental to organisms because they can severely compromise genomic integrity and stability^9^, ^10^.

To address this damage, organisms have evolved various pathways to repair DSBs^11^. In contrast to other prokaryotes, the DSB repair system in mycobacteria is highly complex and diverse, resembling that of *Saccharomyces cerevisiae*, and includes redundant repair components^12^. Therefore, mycobacteria can repair DSBs through multiple distinct pathways. Among these, homologous recombination (HR) is the most accurate DSB repair process but requires an additional intact homologous template. Single‐strand annealing (SSA), another DSB repair mechanism, primarily occurs in the presence of direct repeat sequences flanking the DSB^13^. Mycobacteria also possess the nonhomologous end‐joining (NHEJ) repair mechanism. Unlike the complex NHEJ system in eukaryotic cells, mycobacterial NHEJ consists of only two primary components: Ku, a DNA‐binding protein responsible for end‐binding and end‐bridging, and LigD, a multifunctional enzyme^14^, ^15^. First, the Ku protein binds to the DNA termini, and then LigD facilitates minimal end processing and directly ligates the processed termini without requiring a homologous template, making this repair type error‐prone. Through HR, SSA, or NHEJ repair pathways, mycobacteria effectively repair DSBs^10^. The application of these repair pathways, along with the integration of CRISPR‐Cas systems for gene editing, is becoming increasingly popular in mycobacteria^16^, ^17^, ^18^, ^19^, ^20^.

Methods for the genetic manipulation of mycobacteria are continually being improved^21^. Initially, genetic manipulations such as the expression of *gp60* and *gp61* from the mycobacteriophage Che9c have been accomplished to enhance recombination in *M. smegmatis, M. tuberculosis* ^16^, and *M. abscessus* ^20^. CRISPR‐assisted recombineering has further enabled the efficient construction of marker‐free recombinants in mycobacteria^17^. After its successful implication in *M. smegmatis*, this technique was recently applied successfully for the first time in *M. abscessus* by our group^19^. Additionally, the template‐independent and error‐prone DSB repair mechanism NHEJ presents the potential for genetic manipulation when combined with CRISPR. To date, the CRISPR‐NHEJ gene manipulation approach has proven effective in *M. smegmatis* and *M. tuberculosis*; however, no studies have reported the application of these strategies in *M. abscessus* so far^18^.

In *M. marinum*, *MMAR_4574*, a gene encoding a sugar kinase from the phosphofructokinase B‐type (PfkB) family, is located between the *ku* and *ligD* genes on the chromosome and is referred to as *nrgA* (NHEJ‐related gene A)^18^. The structure of NrgA has recently been elucidated^22^, however, this protein has no homolog in *M. abscessus*. A recent study has shown that in both *M. marinum* and *M. tuberculosis*, mutations in *nrgA* significantly reduced the efficiency of CRISPR‐NHEJ‐based gene editing^18^. This suggests that *nrgA* may play an important role in the NHEJ repair pathway in *M. marinum*. However, the specific mechanisms remain unclear.

To achieve efficient gene knockout using the CRISPR‐NHEJ strategy in *M. smegmatis* and *M. tuberculosis*, it is necessary to regulate their inherent HR systems^18^. This can be accomplished by expressing the negative regulator of *recA*, *recX*, or by directly knocking out the key HR pathway gene, *recA*, to suppress or eliminate HR^23^, ^24^. Under these conditions, a significant increase in gene editing efficiency is observed. Conversely, when the HR repair is not regulated, the efficiency of gene editing using the CRISPR‐NHEJ strategy is extremely low. Based on these findings, researchers have suggested that HR and NHEJ are competitive pathways for the repair of DSBs induced by the CRISPR‐Cas system in mycobacteria^18^.

Here, we report for the first time that gene editing using the CRISPR‐NHEJ strategy is efficient in *M. abscessus*. We also discovered that NrgA, a component of the NHEJ system in *M. marinum*, is indispensable for the NHEJ‐mediated repair of DSBs induced by the CRISPR‐Cas system in *M. abscessus*. Moreover, based on previous research, we investigated the impact of *recX* on the gene editing efficiency of the CRISPR‐NHEJ strategy in *M. abscessus* and *M. smegmatis* and observed different outcomes. We further explored the relationship among the three DSB repair pathways in *M. abscessus*. Our findings indicate a complex interplay among these three repair pathways during the DSB repair process, providing a basis for future studies to further elucidate their specific roles and interactions.

## RESULTS

### Expression of *M. marinum* NHEJ components enhances CRISPR‐Cpf1 gene editing in *M. abscessus*

Compared to the complex NHEJ system in eukaryotes, the NHEJ system in *M. marinum* is relatively simple, consisting of only three components: LigD, Ku, and NrgA. It has been demonstrated that the endogenous NHEJ system in *M. marinum* can effectively repair DSBs induced by the CRISPR‐Cas system, underscoring its potential as a robust repair mechanism^18^. To explore its utility in enhancing gene editing, we first constructed a plasmid, pNHEJ‐Cpf1, which simultaneously expresses CRISPR‐Cpf1 and the complete *M. marinum* NHEJ repair system (Ku, NrgA, LigD) (Figure S1). In this plasmid, Cpf1 expression is induced by anhydrotetracycline (ATc), while the NHEJ components are controlled by their native promoters.

**Figure 1:**
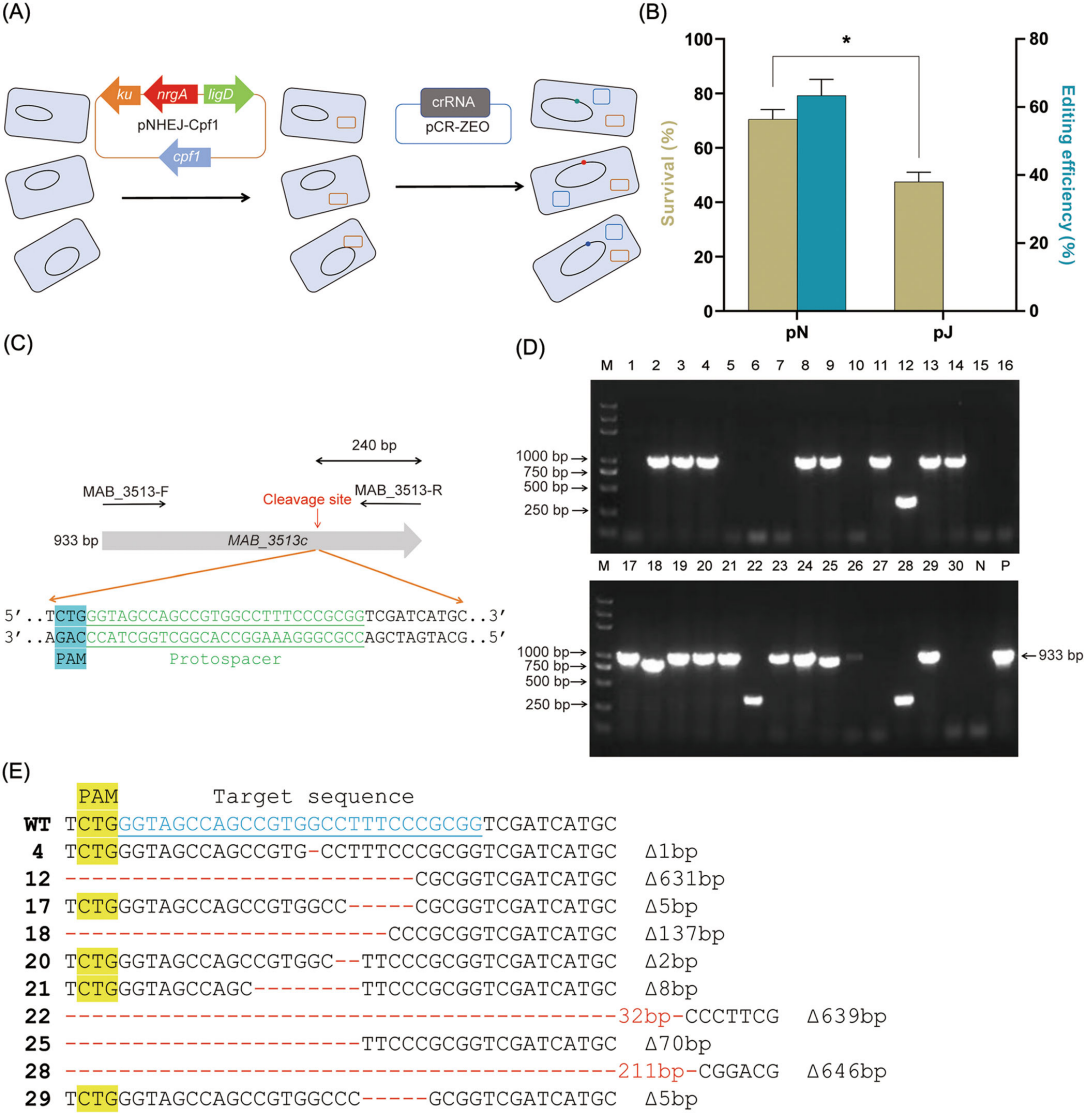
CRISPR‐Cpf1‐NHEJ‐assisted gene editing in *Mycobacterium abscessus*. (A) Schematic of CRISPR‐Cpf1 coupled with the *M. marinum* NHEJ repair in *M. abscessus*. In the first step, Cpf1 and *M. marinum* NHEJ components were expressed in *M. abscessus*. Then, the crRNA‐expressing plasmid pCR‐Zeo was transformed into the cell. (B) Expression of *M. marinum* NHEJ improves the survival rate and enables effective gene editing in *M. abscessus*. The plasmid expressing crRNA targeting *MAB_3513c* was introduced into *M. abscessus* strains harboring pNHEJ‐Cpf1 or pJV53‐Cpf1, respectively. pN, *M. abscessus* cells carrying the pNHEJ‐Cpf1 plasmid. pJ, *M. abscessus* cells carrying the pJV53‐Cpf1 plasmid. Data represent mean ± standard deviation (SD) from three independent experiments. *p* values were determined using Student's unpaired *t*‐test. *p < 0.05. (C) Schematic diagram of the coding sequence, identification primers, cleavage sites, PAM, and protospacer sequence for *MAB_3513c*. (D) Colony PCR results (agarose gel electrophoresis) of randomly selected clones for *MAB_3513c* deletion under the action of the *M. marinum* repair system. Thirty single clones were randomly selected and the *MAB_3513c* gene was amplified using the MAB_3513‐F/R primer pair from (C). N, the PCR result for the negative control Mab^Δ3513c^; P, the PCR result for the positive control of the *M. abscessus* wild‐type (WT) strain. The expected amplicon length is 933 bp. (E) Sequencing results for the *MAB_3513c* target site in selected single colonies. Sequences highlighted with a yellow background indicate the PAM sequences, blue sequences represent the crRNA target sequences, red segments indicate single‐base deletions, and the numbers correspond to the respective mutant strains.

To investigate the effect of the *M. marinum* NHEJ system on gene editing efficiency in *M. abscessus*, we transformed *M. abscessus* cells with either pNHEJ‐Cpf1 or the control plasmid pJV53‐Cpf1. In the absence of acetamide induction, pJV53‐Cpf1 only expresses Cpf1 in *M. abscessus* cells (the plasmids used in this study are listed in Table S1). Following transformation, we introduced a second plasmid, pCR‐ZEO‐3513c, which expresses the crRNA targeting the *MAB_3513c* gene (Figure 1A). This dual‐plasmid system was then used to knock out the *MAB_3513c* gene, and subsequently, the differences in survival rates and gene editing efficiency were observed. After being transformed with the pCR‐ZEO‐3513c plasmid, the bacteria were incubated for 4–5 h, before being plated on 7H11 agar plates containing the appropriate antibiotics and 200 ng/ml ATc. Plates lacking ATc were used as controls. The survival rate was defined as the ratio of colony‐forming units (CFUs) on ATc‐containing plates to those on control plates without ATc. The survival rate of the pNHEJ‐Cpf1 group (71.00% ± 2.83%) was significantly higher than that of the pJV53‐Cpf1 group (47.50% ± 3.54%) (*p* = 0.0156, Figure 1B and the plate photographs for the pNHEJ‐Cpf1 group are shown in Figure S2). This suggests that the expression of the *M. marinum* NHEJ system repaired some of the DSBs caused by CRISPR‐Cpf1, leading to partial colony recovery. We further analyzed the gene editing efficiency of *M. abscessus* obtained through the CRISPR‐NHEJ strategy. Single colonies from the ATc‐containing plates were randomly selected, and the *MAB_3513c* gene was amplified using the primer pair Mab_3513c‐F/R (Figure 1C). The electrophoresis results presented in Figure 1D show that the PCR products from specific single colonies were significantly smaller than those of the wild‐type strain or were completely absent. These outcomes likely resulted from extensive DNA deletions during the NHEJ repair process. However, the sizes of PCR products from certain single colonies were nearly identical to those of the wild‐type strain. This does not necessarily indicate that the target sites remained unchanged, as small alterations of just a few base pairs cannot be easily detected by electrophoresis alone. Therefore, we proceeded with sequencing these single colonies. The sequencing results for altered target sites are shown in Figure 1E (raw sequencing data can be found in Figure S3). In addition to the large fragment deletions discernible from the PCR results, several single colonies, such as colonies 4, 17, 20, 21, and 29, showed only a few base pair of deletions. These DNA fragment deletions, which are not multiples of three, result in frameshift mutations that ultimately lead to partial or complete inactivation of the gene. Statistical analysis revealed that the gene editing efficiency reached 63.35% ± 4.74% for knocking out the *MAB_3513c* gene using the CRISPR‐NHEJ strategy. In contrast, none of the single colonies randomly selected from the pJV53‐Cpf1 group showed any evidence of gene editing (Figure 1B). This indicates that the endogenous NHEJ repair system in *M. abscessus* is not effective at repairing the DSBs caused by CRISPR‐Cpf1. However, when the exogenous *M. marinum* NHEJ system was expressed, it facilitated error‐prone repair of the DSBs induced by CRISPR‐Cpf1.

After successfully knocking out the *MAB_3513c* gene using the CRISPR‐NHEJ strategy, we aimed to assess the broader applicability of this method by targeting additional genes in *M. abscessus*. To date, we have successfully knocked out more than 30 genes in *M. abscessus* (Table S2). Compared to existing methods, this approach has significantly improved the efficiency of generating *M. abscessus* mutants.

### NrgA acts as a crucial factor in repairing CRISPR‐Cas‐induced DSBs via NHEJ in *M. abscessus*

Although *M. abscessus* possesses its own endogenous NHEJ repair system, our findings indicate that this system is ineffective for repairing DSBs induced by the CRISPR‐Cpf1 system. Effective repair of such DSBs in *M. abscessus* requires the introduction of the exogenous NHEJ repair system from *M. marinum*.

**Figure 2:**
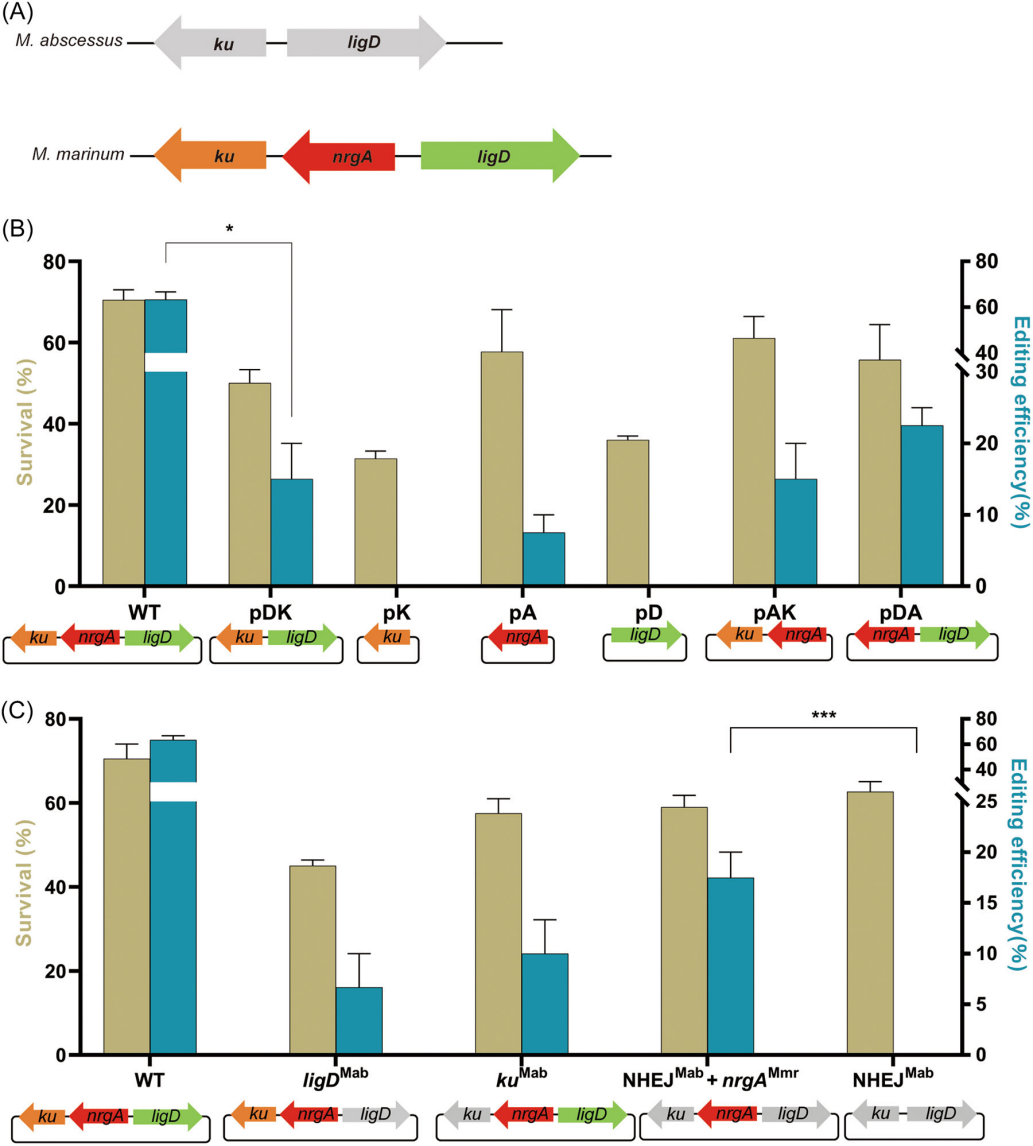
CRISPR‐Cpf1‐NHEJ‐assisted gene editing in *M. abscessus* with different NHEJ components. (A) Schematic of NHEJ components in *M. abscessus* and *M. marinum*. (B) CRISPR‐Cas12a‐NHEJ‐assisted gene editing in *M. abscessus* with certain *M. marinum* NHEJ components. (C) CRISPR‐Cas12a‐NHEJ‐assisted gene editing in *M. abscessus* with hybrid NHEJ components. Plasmids carrying the crRNA were electroporated into *M. abscessus* cells containing plasmids expressing different NHEJ components. Data represent mean ± standard deviation (SD) from three independent experiments. *p* values were determined using Student's unpaired *t‐*test. *p < 0.05; ***p < 0.001.

Compared to *M. abscessus*'s NHEJ repair system, the *M. marinum* NHEJ system includes not only LigD and Ku but also NrgA (Figure 2A). In *M. marinum*, NrgA has been shown to improve the efficiency of CRISPR‐NHEJ‐mediated gene editing^18^. Thus, two factors may account for the necessity of introducing the *M. marinum* NHEJ system into *M. abscessus*: differences in LigD and Ku between the two species and the presence of the additional NrgA factor in *M. marinum*.

To investigate this, we constructed plasmids containing various combinations of *M. marinum* NHEJ components, specifically, *ku*, *nrgA*, *ligD*, *ku+nrgA*, *nrgA+ligD*, or *ligD+ku* along with Cpf1. These plasmids were designated as pK‐Cpf1, pA‐Cpf1, pD‐Cpf1, pAK‐Cpf1, pDA‐Cpf1, and pDK‐Cpf1, respectively. We then analyzed the survival rates and gene editing efficiencies in *M. abscessus* strains carrying these plasmids following CRISPR‐NHEJ gene editing.

Compared to the pNHEJ‐Cpf1 group, *M. abscessus* strains harboring plasmids with only partial *M. marinum* NHEJ components showed reduced survival rates, indicating a decline in the number of single colonies recovered through NHEJ repair (Figure 2B). Further analysis of the gene editing efficiency revealed that strains co‐expressing *M. marinum ku* and *ligD* showed a significant reduction in gene editing efficiency compared to the pNHEJ‐Cpf1 group, achieving only 15.00% ± 7.07% (*p* = 0.0151). Strains expressing either *ku* or *ligD* alone failed to produce any edited colonies.

Interestingly, in the group expressing only *M. marinum nrgA*, approximately 7.50% ± 3.54% of single colonies showed successful gene editing. This suggests that the endogenous NHEJ system of *M. abscessus* may show limited efficiency in repairing DSBs caused by the CRISPR‐Cpf1 system in the presence of NrgA. Compared to the NrgA group, in groups co‐expressing *M. marinum nrgA* with either *ku* or *ligD*, gene editing efficiencies increased to 15.00% ± 7.07% and 22.50% ± 3.53%, respectively. These results indicate that NrgA facilitates the repair process and that exogenous Ku and LigD can partially complement the endogenous NHEJ system in *M. abscessus* (Figure 2B).

To further investigate the differences in survival rates and gene editing efficiencies, we replaced *M. marinum* NHEJ components with their counterparts from *M. abscessus*. Plasmids were constructed containing various combinations of *M. abscessus* and *M. marinum* NHEJ components: pNHEJ‐Cpf1 (*ligD* ^Mab^), pNHEJ‐Cpf1 (*ku* ^Mab^), pNHEJ‐Cpf1 (NHEJ^Mab^ + *nrgA* ^Mmr^), and a plasmid containing only *M. abscessus* NHEJ components without *M. marinum nrgA*, pNHEJ‐Cpf1 (NHEJ^Mab^). Gene editing experiments were performed in *M. abscessus* strains carrying these plasmids.

The survival rates and gene editing efficiencies in groups carrying these plasmids were consistently lower than those observed in strains expressing the pNHEJ‐Cpf1 plasmid containing the complete *M. marinum* NHEJ system (Figure 2C). Notably, the gene editing efficiency in the group where both *ligD* and *ku* of *M. marinum* were replaced with *ligD* and *ku* of *M. abscessus* (17.50% ± 3.54%) was higher than that in groups where only *ligD* (6.65% ± 4.72%) or *ku* (10.00% ± 4.71%) from *M. marinum* was replaced with their respective *M. abscessus* components. These results indicate that high gene knockout efficiency requires LigD and Ku to originate from the same bacterial species. This also explains the slightly reduced gene editing efficiency observed in *M. abscessus* strains expressing mixed NHEJ components compared to those expressing only *M. marinum* components. The decrease in NHEJ repair efficiency observed with increasing proportions of mixed NHEJ components highlights the importance of compatibility between LigD and Ku from the same species (Figure 2B,C). Furthermore, no single colonies showing targeted gene editing were observed in the NHEJ^Mab^ group (*p* < 0.001), in contrast to the NHEJ^Mab^ + *nrgA* ^Mmr^ group, further confirming the critical role of *nrgA* ^Mmr^, which is absent in *M. abscessus*, in facilitating NHEJ repair and achieving efficient gene editing in *M. abscessus*.

### Inhibition of HR differentially affects CRISPR‐NHEJ gene editing efficiency in *M. smegmatis* and *M. abscessus*

Previous studies have demonstrated that inhibiting HR in *M. smegmatis* significantly enhances the efficiency of CRISPR‐NHEJ editing, suggesting a competitive relationship between HR and NHEJ during the DSB repair process in this organism^18^. Conversely, in *M. abscessus*, we achieved efficient gene editing using the CRISPR‐NHEJ strategy without the additional expression of *recX*. The contrasting results between *M. smegmatis* and *M. abscessus* prompted further investigation.

**Figure 3:**
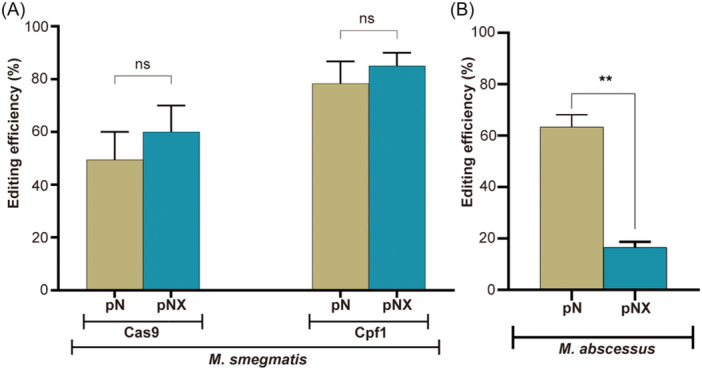
Effects of overexpressing the *recX* gene on the editing efficiency of *M. abscessus* and *M. smegmatis*. (A) Effects of overexpressing the *recX* ^*Msm*^ gene on the editing efficiency of *M. smegmatis*. The plasmid expressing sgRNA targeting *MSMEG_1946* and Cas9 sequence was introduced into *M. smegmatis* strains harboring pNHEJ‐SacB or pNHEJ‐SacB(*recX* ^Msm^) and the plasmid expressing crRNA targeting *MSMEG_1946* was introduced into *M. smegmatis* strains harboring pNHEJ‐Cpf1 or pNHEJX‐Cpf1(*recX* ^Msm^), respectively. (B) Effects of overexpressing the *recX* ^*Mab*^ gene on the CRISPR‐Cas12a‐NHEJ editing efficiency of *M. abscessus*. pN, plasmid with no *recX* but including the sequence for the corresponding CRISPR effector proteins. pNX, plasmid with *recX* and including the sequence for the corresponding CRISPR effector proteins. Data represent mean values ± SD from three independent experiments. *p* values were determined using Student's unpaired *t‐*test. ns, not significant; **p < 0.01.

We first evaluated the impact of *recX* on the CRISPR‐NHEJ gene editing efficiency in *M. smegmatis*. Specifically, we assessed NHEJ repair of DSBs induced by two different CRISPR effector proteins (Cas9 and Cpf1) under the influence of *recX* in *M. smegmatis*. When using Cas9 as the effector protein, the gene editing efficiency in the group without *recX* expression was 49.45% ± 14.92%, while the gene editing efficiency with *recX* was 60.00% ± 14.14%, showing no significant difference (*p* = 0.5434, Figure 3A). In the case of using Cpf1 as the effector protein, the gene editing efficiency in the group without *recX* was 78.35% ± 11.81%, and with *recX*, it was 85.00% ± 7.07%, similarly showing no significant difference (*p* = 0.5650, Figure 3). These findings indicate that in *M. smegmatis*, we can achieve high‐efficiency gene editing without the need for additional *recX* expression or manipulation of the host's HR system, which is significantly different from previously reported results^18^.

In *M. abscessus*, our preliminary research also showed that high‐efficiency CRISPR‐NHEJ‐based gene editing does not require additional *recX* expression. We further observed the effect of overexpressing *recX* on the gene editing efficiency of the CRISPR‐Cpf1‐NHEJ strategy in *M. abscessus*. Surprisingly, overexpressing *recX*, the gene editing efficiency significantly decreased to just 16.55% ± 2.19%, approximately 1/4 of the original efficiency (Figure 3B). This result strongly suggests that, in *M. abscessus*, inhibiting HR negatively impacts the efficiency of NHEJ‐mediated repair during the DSB repair process.

### DSB repair pathways—NHEJ, HR, and SSA in *M. abscessus*—exist in a dynamic balance

In mycobacteria, the DSB repair pathways include not only HR and NHEJ but also SSA. While previous studies have explored the influence of SSA on HR efficiency in mycobacteria^25^, the potential effect of SSA on NHEJ has not been adequately investigated. To address this gap, we examined whether disrupting HR or SSA impacts the NHEJ‐dependent gene editing efficiency in *M. abscessus*. Using CRISPR‐assisted recombineering as described in our previous study^19^, we constructed selection‐marker‐free, in‐frame deletion knockout strains targeting the *recA*, *ruvB*, *recC*, *recO*, *ligD*, and *ku* genes. RecA plays a crucial role in HR, particularly at the initiation stage of repair, whereas RuvB facilitates the branch migration of Holliday junctions, ensuring accurate DSB repair^26^, ^27^, ^28^. The RecBCD complex, including RecC, is essential for SSA and is independent of RecA‐mediated HR^13^. Additionally, RecO participates in both HR and SSA pathways^29^, ^30^. LigD and Ku are two core components of the endogenous NHEJ repair system in *M. abscessus*.

**Figure 4:**
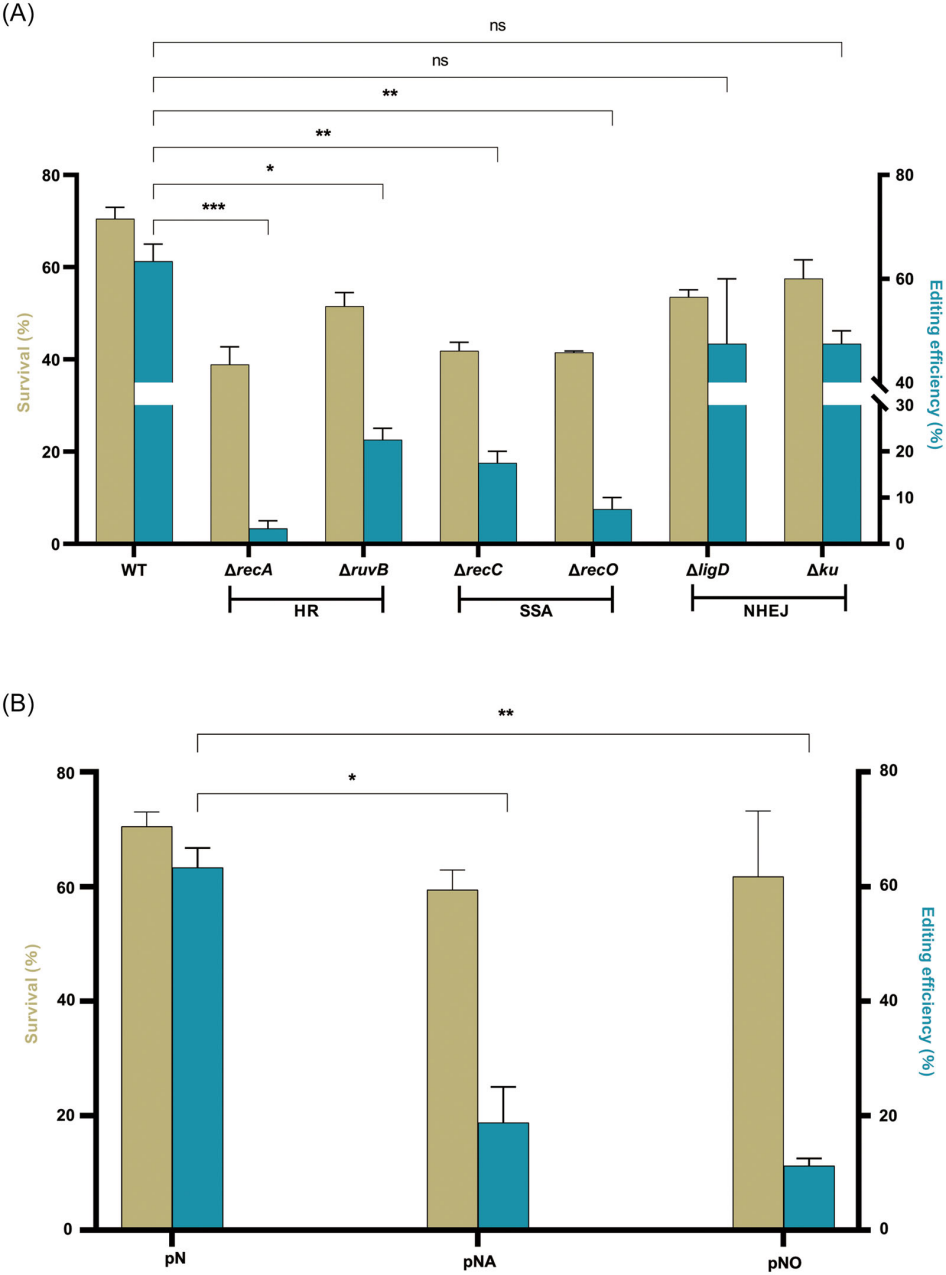
Survival rates and CRISPR‐Cpf1‐NHEJ‐assisted gene editing efficiencies in *M. abscessus* and its derivatives. (A) Survival rates and gene editing efficiencies in *M. abscessus* with the key genes in HR or SSA knocked out. Plasmids carrying the crRNA were electroporated into *M. abscessus* and its derivatives. (B) A significant decrease in gene editing efficiency in *M. abscessus* induced by overexpressing *recA* and *recO* gene. pN, *M. abscessus* carrying the pNHEJ‐Cpf1 plasmid; pNA, *M. abscessus* carrying the pNHEJA‐Cpf1 plasmid (overexpressing *recA*); pNO, *M. abscessus* carrying the pNHEJO‐Cpf1 plasmid (overexpressing *recO*). Data represent the mean ± SD from three independent experiments. *p* values were determined using Student's unpaired *t‐*test. ns, not significant. *p < 0.05; **p < 0.01; ***p < 0.001. HR, homologous recombination; SSA, single strand annealing.

We performed gene editing experiments in these endogenous HR, SSA, or NHEJ repair‐deficient strains to observe the effects of these repair pathway disruptions on gene editing efficiency. The gene editing efficiencies were 3.33% ± 2.88%, 22.50% ± 3.54%, 17.50% ± 3.54%, and 7.50% ± 3.54% in the *recA*, *ruvB*, *recC,* or *recO* knockout strains, respectively (Figure 4A). As expected, the gene editing efficiency in the *recA* knockout strain was significantly lower than that in the wild‐type strain (*p* = 0.0004). Similarly, the gene editing efficiencies in the *ruvB*, *recC,* or *recO* gene knockout strains were significantly reduced compared to the wild‐type strain (*p* = 0.0103, *p* = 0.0082, and *p* = 0.0056, respectively, Figure 4A). All these results confirmed that disruption of the HR or SSA repairing pathways would decrease the CRISPR‐Cas12a‐assisted NHEJ‐mediated gene editing efficiency.

In contrast, the *ligD* and *ku* knockout strains showed gene editing efficiencies of 47.50% ± 17.68% and 47.50% ± 3.54%, respectively. Although the difference in editing efficiency between these strains and the wild‐type strains was insignificant, there was still a slight decrease (Figure 4A). This aligns with our previous findings, which demonstrated that the endogenous NHEJ system in *M. abscessus* can manage low‐efficiency DSB repair when only *nrgA* is introduced (Figure 2B). Therefore, knocking out *ligD* or *ku* eliminated this endogenous NHEJ‐dependent repair capacity, resulting in a slight decrease in gene editing efficiency compared to the wild‐type strain.

Interestingly, editing efficiencies of the knockout strains ranked as follows: *recA* (HR) *≤ recO* (HR & SSA) *≤ recC* (SSA) *≤ ruvB* (HR). Significant differences in editing efficiencies were observed between *recA* and *recC* (*p* = 0.0156) and between *recA* and *ruvB* (*p* = 0.0067). This suggests that NHEJ‐dependent gene editing efficiency is differentially influenced by the specific genes within the HR and SSA DSB repair pathways. Genes involved in the initiation of HR repair may have a greater impact on editing efficiency compared to those involved later in the process. Furthermore, genes that participate in HR pathways may exert a stronger influence on editing efficiency than those affecting only SSA. While *recO* might influence both HR and SSA, it primarily affects a branch pathway of HR, unlike *recA*, which plays a central role in HR^28^, ^30^, ^31^.

Based on the above results, we found that inhibiting the HR and SSA pathways in *M. abscessus* leads to a significant decrease in NHEJ repair efficiency. This raises the question of whether enhancing the HR or SSA pathways could improve NHEJ repair efficiency. To test this hypothesis, we further overexpressed the *recA* and *recO* genes and repeated the gene editing experiment. Contrary to our expectations, overexpressing either *recA* or *recO* did not enhance the NHEJ‐dependent gene editing efficiency. Instead, in *recA* and *recO* overexpressing groups, the gene editing efficiency significantly decreased by 18.75% ± 8.84% and 11.25% ± 1.77%, respectively, compared to the wild‐type strain (Figure 4B).

These results suggest that either excessive enhancement or inhibition of HR or SSA pathways leads to a decrease in NHEJ‐based editing efficiency. This decline may be attributed to the disruption of the delicate balance among HR, SSA, and NHEJ repair pathways. In summary, rather than functioning as isolated or competing mechanisms, HR, SSA, and NHEJ interact dynamically during DSB repair, maintaining a state of equilibrium crucial for efficient repair processes.

## DISCUSSION

*M. abscessus*, a prevalent NTM, has emerged as a significant global health concern due to its increasing incidence and intrinsic resistance to many commonly used antibiotics, severely limiting the effectiveness of current treatment regimens, which makes *M. abscessus*‐induced infections extremely difficult to treat and poses a serious threat to public health^5^, ^32^. The growing issue of drug resistance not only highlights the limitations of existing antimicrobial therapies but also underscores the urgent need to accelerate the development of novel treatment strategies. A comprehensive understanding of the resistance mechanisms and drug targets of *M. abscessus* is essential for designing more effective drugs and therapeutic regimens. Therefore, developing advanced genetic manipulation tools to rapidly and efficiently generate *M. abscessus* gene knockout strains is of paramount importance^33^, ^34^.

Traditional recombineering‐based methods for gene editing in *M. abscessus* are hindered by several limitations, including low efficiency, reliance on homologous templates, and the potential for introducing genomic scars. These constraints restrict the rapid generation of large numbers of knockout mutants^16^. In contrast, the CRISPR‐Cas12a‐NHEJ strategy that we developed offers a marked improvement, achieving a gene editing efficiency exceeding 60% for the *MAB_3513c* gene and ranging from 40% to 90% across more than 30 target genes. This approach accelerates the pace of research on *M. abscessus* by overcoming the limitations of conventional methods. Importantly, the approach generated mutants with deletions of varying lengths around the target site, facilitating the selection of strains with specific mutations according to experimental requirements.

Our findings also underscore the critical role of NrgA in the NHEJ repair process. Unlike *M. marinum*, which possesses LigD, Ku, and NrgA, the NHEJ system in *M. abscessus* lacks NrgA. A previous study has shown that NrgA enhances NHEJ repair efficiency in both *M. marinum* and *M. tuberculosis*, indicating its important role in repairing CRISPR‐Cas‐induced DSBs^18^. Our experiments confirmed this hypothesis, demonstrating that NrgA can synergize with the endogenous NHEJ system in *M. abscessus* to enable low‐efficiency gene editing. Moreover, combining NHEJ components from different sources (e.g., *M. abscessus* LigD and Ku with *M. marinum* NrgA) resulted in significantly decreased editing efficiency compared to expressing the complete *M. marinum* NHEJ system. This highlights the importance of compatibility among DSB repair components in achieving efficient NHEJ repair.

Another important aspect to consider is the survival rate. When the complete *M. marinum* NHEJ repair system was introduced, the colony recovery rate significantly increased compared to the pJ group (Figure 1B). However, changes in survival rates did not directly correspond to changes in gene editing efficiency. Notably, in *M. abscessus*, no modifications at the target sites were observed before the introduction of the *M. marinum* NHEJ repair system, regardless of whether DSB repair was mediated by endogenous HR, SSA, or NHEJ. Upon introducing the NHEJ repair system or its components, both the independent repair role of exogenous NHEJ and its interactions with endogenous DSB repair pathways must be considered. This complexity aligns with findings from previous studies, which have shown that introducing mycobacterial NHEJ systems into mycobacteria or other bacteria induces interactions between exogenous NHEJ and endogenous repair mechanisms^18^, ^35^. Under the influence of the exogenous complete *M. marinum* NHEJ system, the repair process originally managed by endogenous DSB repair becomes target site‐modifiable, resulting in increased gene editing efficiency. This complexity might contribute to the lack of a strict correlation between survival rates and gene editing efficiency.

Interestingly, certain groups show high survival rates despite low gene editing efficiency (Figure 2B). This observation is consistent with previous studies where gene editing efficiency varied significantly even among groups with similar survival rates^18^. Groups expressing only partial NHEJ components from *M. marinum* or *M. abscessus* likely experienced enhanced survival due to these components supporting endogenous DSB repair, but effective gene editing was not achieved. Additionally, the stability of the CRISPR‐Cas system itself may influence these outcomes. Mutations in the CRISPR effector protein sequence or the sgRNA cassette sequence could enable colony survival without introducing modifications at the target site. This hypothesis is supported by a previous study of the CRISPR‐NHEJ strategy in *Escherichia coli* ^36^. In future studies, sequencing the CRISPR effector protein genes and sgRNA cassettes from surviving colonies could validate this hypothesis.

A key finding of this study is the dynamic balance among the three DSB repair pathways—HR, SSA, and NHEJ—in *M. abscessus*. Unlike previous studies that suggested competition among these pathways^18^, ^25^, our results indicate potential synergistic interactions. Initially, we observed that disrupting HR did not affect the CRISPR‐Cas12a‐ or CRISPR‐Cas9‐assisted NHEJ gene editing efficiency in *M. smegmatis* (Figure 3A), a finding consistent with a previous study showing that HR disruption does not increase the NHEJ‐mediated DSB repair efficiency in *M. smegmatis* ^13^. In *M. abscessus*, our primary results indicate that the CRISPR‐NHEJ strategy can be successfully applied for efficient gene editing without inhibiting the HR pathway. However, further investigation revealed that HR‐ or SSA‐deficient *M. abscessus* strains showed significantly reduced NHEJ‐based gene‐editing efficiency, suggesting a potential synergistic interaction between HR, SSA, and NHEJ. Similar findings have been reported in *Halomonas bluephagenesis*, where HR inhibition led to a marked reduction in NHEJ‐mediated gene editing efficiency^35^.

**Figure 5:**
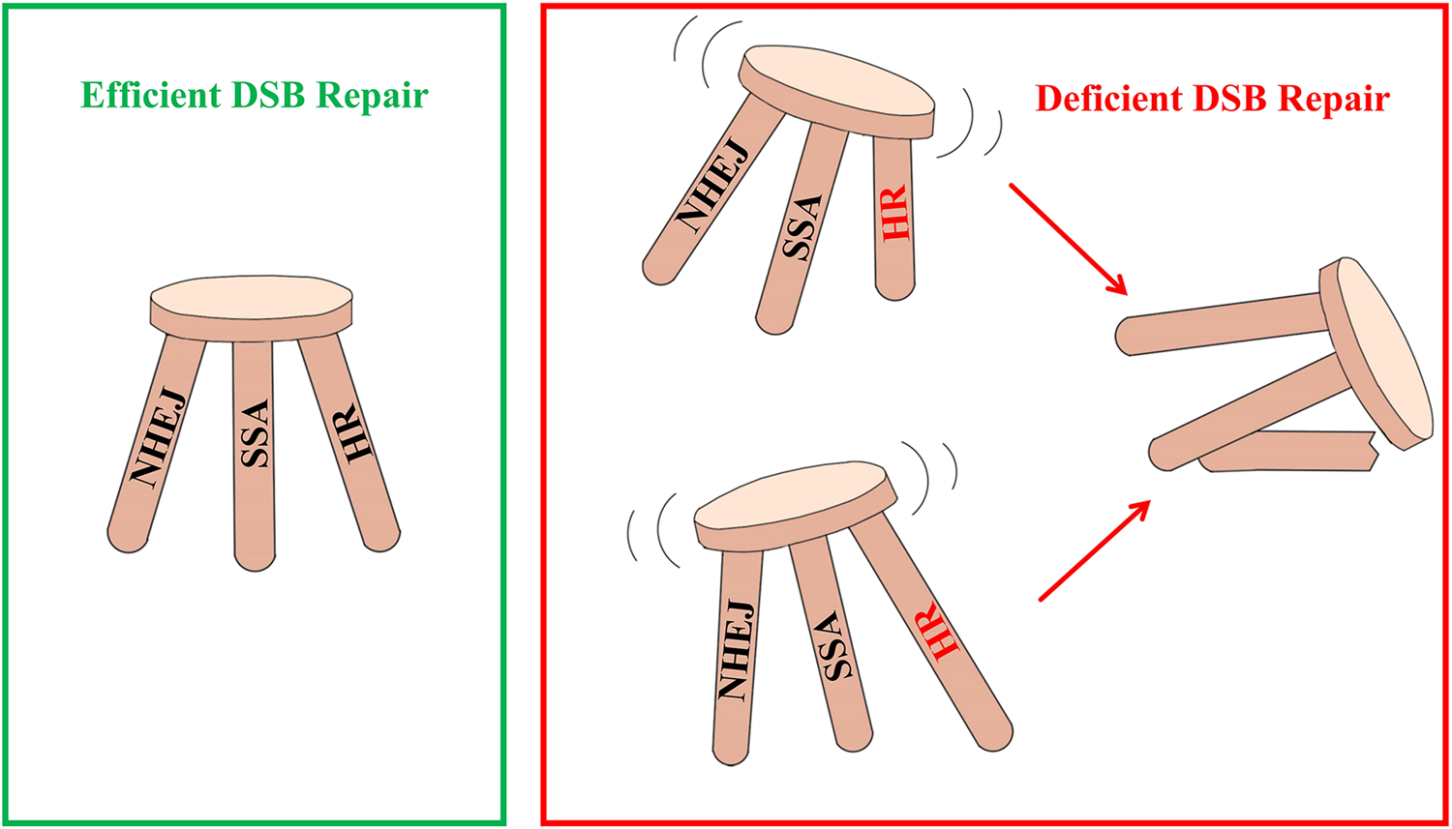
A simplified illustration depicts the hypothesis regarding the interplay among the three DSB repair pathways. Like a three‐legged stool, efficient DSB repair necessitates a harmonious balance among HR, SSA, and NHEJ. Disruption to this equilibrium, exemplified by an imbalance in HR strength—whether too weak or excessively potent—compromises the overall efficacy of DSB repair.

Interestingly, overexpression of key HR or SSA pathway genes, such as *recA* or *recO*, in *M. abscessus* also resulted in a significant decrease in NHEJ efficiency. These results indicate that excessive activity of the HR or SSA pathways can inhibit NHEJ repair, highlighting the importance of a delicate balance among the three DSB repair pathways. We propose that HR, SSA, and NHEJ in *M. abscessus* function in a dynamic equilibrium, akin to a three‐legged stool: efficient DSB repair is achieved only when all three pathways are balanced, while disruption of this balance leads to inefficient DSB repair (Figure 5). Future studies could explore the use of promoters with varying strengths to modulate HR or SSA activity, providing further insights into how these pathways influence NHEJ repair efficiency.

The structure of DSB ends also likely influences the interactions among the three pathways and impacts repair outcomes. For instance, Cpf1 generates DSBs with a 5′ overhangs, whereas Cas9 produces blunt ends^7^. The pathway and outcome of NHEJ repair of DSBs depend on the structure of the broken DNA ends^37^. The processing of different types of DNA ends may involve complex interactions among HR, SSA, and NHEJ. These differences in DNA end structures may affect the preferences and interactions among HR, SSA, and NHEJ repair pathways^13^. All these factors could potentially affect the interactions among the three DSB repair systems. Investigating these interactions further will enhance our understanding of their roles in CRISPR‐assisted gene editing across various mycobacterial species.

In conclusion, we successfully demonstrated the application of the CRISPR‐Cas12a‐NHEJ strategy for efficient gene editing in *M. abscessus* for the first time. Our findings highlight the crucial role of NrgA in the NHEJ repair process and reveal the complex interactions among different repair pathways in *M. abscessus*. This discovery not only challenges previously established perspectives but also provides new insights for advancing genetic manipulation in *M. abscessus* and other mycobacteria. By deepening our understanding of these repair mechanisms, this work lays the foundation for developing more effective gene editing tools, ultimately contributing to research efforts aimed at combating antibiotic resistance and infectious diseases.

## MATERIALS AND METHODS

### Strains, media, and growth conditions

The *E. coli* Trans1‐T1 strain grown in LB medium was used for molecular cloning and plasmid manipulation. *M. abscessus* GZ002 (NCBI GenBank accession number: CP034181)^38^, ^39^ and its derived strains along with *M. smegmatis* MC^2^ 155 were preserved by our laboratory. Liquid cultures of *M. abscessus* and *M. smegmatis* were grown in 7H9 medium supplemented with 10% oleic acid‐albumin‐dextrose‐catalase (OADC) and 0.05% Tween80, while the solid cultures were grown on 7H11 medium supplemented with 10% OADC and 0.05% Tween80 following electroporation. The working concentrations of anhydrotetracycline, kanamycin, and zeocin were 200 ng/ml, 100 μg/ml, and 30 μg/ml for *M. abscessus* and 50 ng/ml, 50 μg/ml, and 30 μg/ml for *M. smegmatis*, respectively.

### Construction of plasmids

The primers used for plasmid construction in this study are listed in Table S3. The plasmids related to the CRISPR‐Cpf1‐NHEJ gene editing system included pCR‐Zeo, pNHEJ‐Cpf1, pDK‐Cpf1, pK‐Cpf1, pA‐Cpf1, pD‐Cpf1, pAK‐Cpf1, pDA‐Cpf1, pNHEJ‐Cpf1 (*ligD* ^Mab^), pNHEJ‐Cpf1 (*ku* ^Mab^), pNHEJ‐Cpf1 (NHEJ^Mab^+*nrgA* ^Mmr^), pNHEJ‐Cpf1 (NHEJ^Mab^), pNHEJX^Mab^‐Cpf1, and pNHEJX^Msm^‐Cpf1. The plasmids related to the CRISPR‐Cas9‐NHEJ gene editing system included pZEO2085, pNHEJ‐SacB, and pNHEJ‐SacBX^Msm^. The *cpf1* coding sequence was derived from the plasmid pJV53‐Cpf1. The NHEJ components of *M. abscessus* and *M. marinum* as well as *recX* from *M. abscessus* and *M. smegmatis* or *recA* and *recO* from *M. abscessus* were amplified using the corresponding primers and ligated into the plasmid backbone containing *cpf1* through recombination methods. The crRNA or sgRNA sequences were synthesized as primer pairs, annealed, and ligated into the linearized pCR‐Zeo or pZEO2085 plasmid.

### Gene editing in *M. abscessus* and *M. smegmatis*

*M. abscessus* and its derivative strains or *M. smegmatis* carrying the respective plasmids were cultured in shake flasks containing 40 ml of complete 7H9 medium at 37°C, 200 rpm, until the OD_600_ = 0.8−1.0. The bacterial cultures were then harvested for competent cell preparation as previously described^40^. About 500 ng of the plasmid expressing guide RNA (primers for constructing crRNA are listed in Table S1) was mixed with 100 μl of competent cells, transferred to a 2 mm electroporation cuvette, and incubated on ice for 10 min. Electroporation was performed using a voltage of 2500 V, a capacitance of 25 μF, and a resistance of 1000 Ω. After electroporation, 2 ml of 7H9 medium was added to the electroporation cuvette to resuspend the bacteria, which was then transferred to a 50 ml tube and incubated at 30°C for 4−5 h to facilitate recovery. Then, cultures were plated separately on 7H11 agar supplemented with the appropriate antibiotics and ATc and incubated at 30°C. The editing efficiency was calculated by measuring the proportion of colonies with mutations at the target site among the total randomly selected clones. The survival rate was defined as the ratio of the number of CFUs obtained from plates containing ATc to the number of CFUs obtained from plates without ATc. For each assessment, at least 20 colonies were randomly selected for PCR (primers are shown in Table S1) and sequencing analysis. Colonies that either lacked the target bands in PCR or showed sequencing results that deviated from the wild‐type sequence were identified as edited.

## AUTHOR CONTRIBUTIONS

**Sanshan Zeng**: Conceptualization (equal); data curation (lead); formal analysis (lead); investigation (lead); methodology (lead); writing‐original draft (lead); writing—review and editing (lead). **Yanan Ju**: Conceptualization (equal) and investigation (equal); **Md Shah Alam**: Conceptualization (equal) and investigation (equal); **Ziwen Lu**: Methodology (equal) and software (equal); **H.M. Adnan Hameed**: Writing—original draft (supporting) and writing—review (lead); **Lijie Li**: Methodology (supporting) and software (supporting); **Xirong Tian**: Validation (supporting) and formal analysis (supporting); **Cuiting Fang**: Validation (supporting) and formal analysis (supporting); **Xiange Fang**: Validation (supporting) and investigation (supporting); **Jie Ding**: Validation (supporting) and investigation (supporting); **Xinyue Wang**: Validation (supporting) and writing—review (equal); **Jinxing Hu**: Validation (supporting) and writing—review (supporting); **Shuai Wang**: Project administration (lead) and funding acquisition (lead); **Tianyu Zhang**: Project administration (lead) and funding acquisition (lead)

## ETHICS STATEMENT

This study did not involve any human participant or animal subject.

## CONFLICT OF INTERESTS

The authors declare no conflict of interests.

## Supporting information

Supporting information.

## Data Availability

The authors confirm that the data supporting the findings of this study are available within the article and its supporting information materials.
