# Supplementary material for: A CRISPR‐nonhomologous end‐joining‐based strategy for rapid and efficient gene disruption in Mycobacterium abscessus
Source: mLife. 2025 Apr 23;4(2):169–80. doi: 10.1002/mlf2.70007 (PMC12042118; doi:10.1002/mlf2.70007)
Supplement: Supplementary file 1 — Supporting information. [file MLF2-4-169-s001.docx]

Supplementary Materials

**A CRISPR-nonhomologous end-joining-based strategy for rapid and efficient gene disruption in *Mycobacterium abscessus***

Sanshan Zeng,^1,2,3,4^ Yanan Ju,^1,2,3,5^ Md Shah Alam,^1,2,3,4^ Ziwen Lu,^1,2,3,4^ H.M. Adnan Hameed,^1,2,3^ Lijie Li,^1,2,3,5^ Xirong Tian,^1,2,3,4^ Cuiting Fang,^1,2,3,4,6^ Xiange Fang,^1,2,3,4^ Jie Ding,^1,2,3,7^ Xinyue Wang,^6,8^ Jinxing Hu,^6^ Shuai Wang,^1,2,3,6*^ Tianyu Zhang ^1,2,3,4,5,6,7,8*^

^1^ State Key Laboratory of Respiratory Disease, Guangzhou Institutes of Biomedicine and Health, Chinese Academy of Sciences, Guangzhou, China

^2^ Guangdong-Hong Kong-Macao Joint Laboratory of Respiratory Infectious Diseases, Guangzhou Institutes of Biomedicine and Health, Chinese Academy of Sciences, Guangzhou, China

^3^ China-New Zealand Joint Laboratory on Biomedicine and Health, Guangzhou Institutes of Biomedicine and Health, Chinese Academy of Sciences, Guangzhou, China

^4^ University of Chinese Academy of Sciences, Beijing, China

^5^ School of Basic Medical Sciences, Division of Life Science and Medicine, University of Science and Technology of China, Hefei, China

^6^ State Key Laboratory of Respiratory Disease, Guangzhou Chest Hospital, Guangzhou, China

^7^ Institute of Physical Science and Information Technology, Anhui University, Hefei, China

^8^ Guangzhou National Laboratory, Guangzhou, China

^*^ Corresponding authors: Shuai Wang, E.mail: [wshuai1@163.com,](mailto:wshuai1@163.com,) Tel: +86-20-32015266; Tianyu Zhang, E.mail: [zhang_tianyu@gibh.ac.cn](mailto:zhang_tianyu@gibh.ac.cn), Tel: +86-20-32015270.

**Figure S1.** Map of plasmid pNHEJ-Cpf1 developed for CRISPR-Cpf1-NHEJ-assisted gene editing in *M. abscessus* and *M. smegmatis*. *ligD*, *nrgA*, and *ku* compose the *M. marinum* NHEJ element. *kanR*, confers resistance to kanamycin in bacteria. *tetR*, encodes the tetracycline repressor protein, which regulates the expression of gene downstream of the tetracycline operator. Cpf1, a CRISPR-associated endonuclease used for cleaving specific DNA sequences.


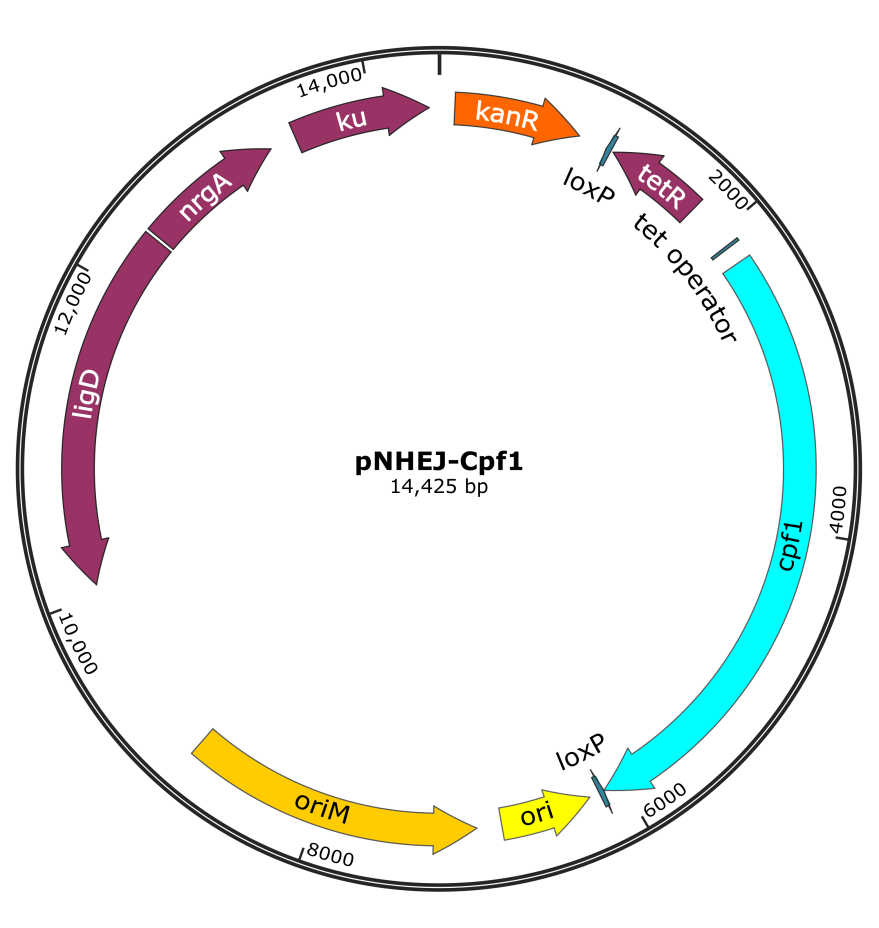


**Figure S2.** Growth of *M. abscessus* carrying pNHEJ-Cpf1 with pCR-ZEO-3513c, plated on media with or without ATc. A: Growth of undiluted bacterial culture on plates without ATc. B: Growth of undiluted bacterial culture on plates containing 200 ng/mL ATc. C: Growth of 1,000-fold diluted bacterial culture on plates without ATc. D: Growth of 1,000-fold diluted bacterial culture on plates containing 200 ng/mL ATc.

**
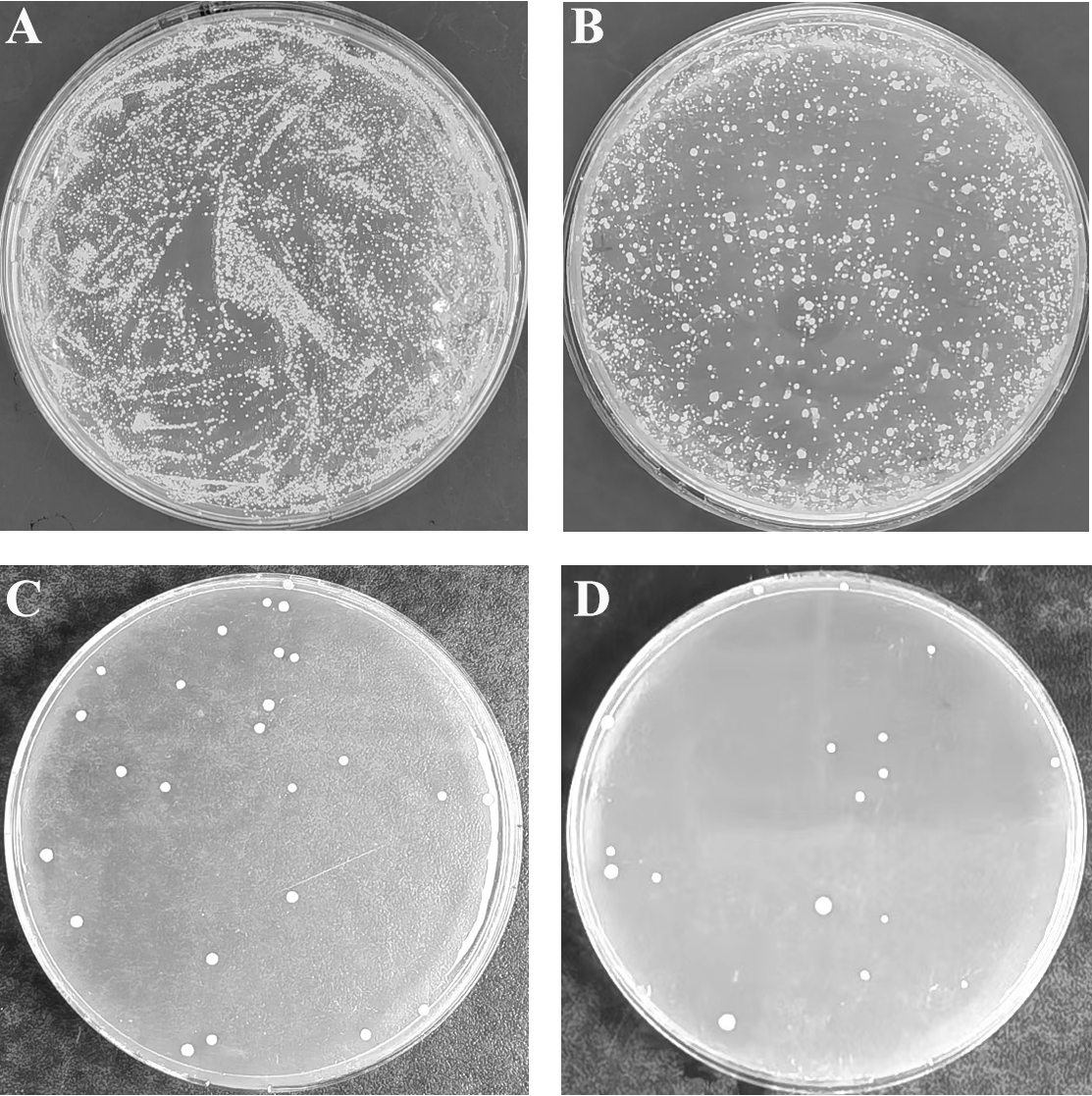
**

**Figure S3. Raw sequencing data in FIG 1E.**

**
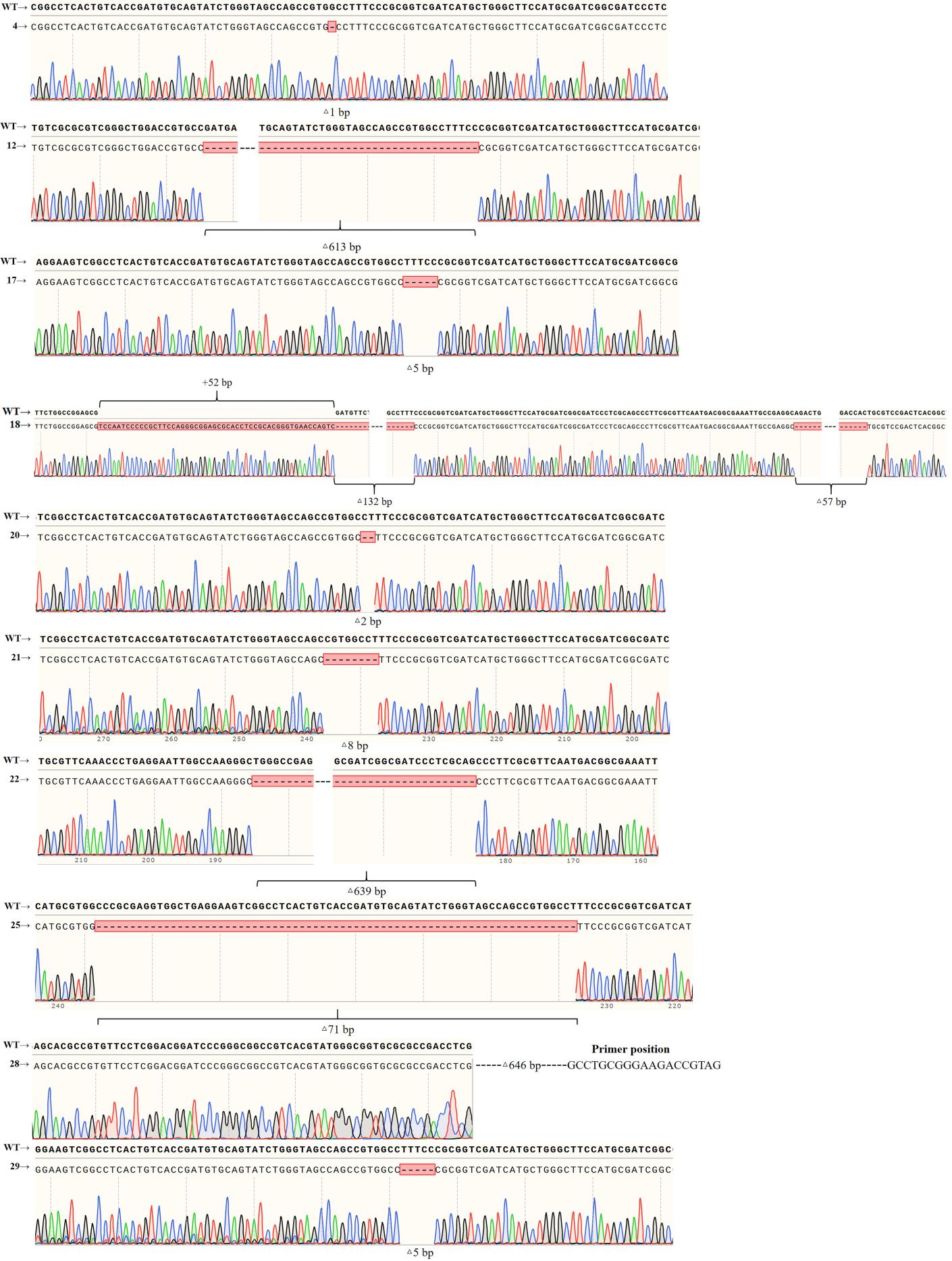
**

**Table S1.** Plasmids used in this study.

| **Plasmid** | **Relevant characteristics** | **Source** |
| --- | --- | --- |
| pJV53-Cpf1 | Used for gene knockout in *M. abscessus* and *M. smegmatis* based on the CRISPR-Cpf1-HR strategy; Cpf1 is expressed under a tetracycline-inducible system, while *gp60/61* is expressed under acetamide induction; Kanamycin resistance. | [1] |
| pNHEJ-Cpf1 | Used for gene knockout in *M. abscessus* and *M. smegmatis* based on the CRISPR-Cpf1-NHEJ strategy; Cpf1 is expressed under a tetracycline-inducible system, while *M. marinum* NHEJ is expressed constitutively; Kanamycin resistance. | This study |
| pDK-Cpf1 | pNHEJ-Cpf1-derived plasmid; Cpf1 is expressed under a tetracycline-inducible system, while *M. marinum* *ligD* and *ku* are expressed constitutively; Kanamycin resistance. | This study |
| pK-Cpf1 | pNHEJ-Cpf1-derived plasmid; Cpf1 is expressed under a tetracycline-inducible system, while *M. marinum* *ku* is expressed constitutively; Kanamycin resistance. | This study |
| pA-Cpf1 | pNHEJ-Cpf1-derived plasmid; Cpf1 is expressed under a tetracycline-inducible system, while *M. marinum* *nrgA* is expressed constitutively; Kanamycin resistance. | This study |
| pD-Cpf1 | pNHEJ-Cpf1-derived plasmid; Cpf1 is expressed under a tetracycline-inducible system, while *M. marinum* *ligD* is expressed constitutively; Kanamycin resistance. | This study |
| pDA-Cpf1 | pNHEJ-Cpf1-derived plasmid; Cpf1 is expressed under a tetracycline-inducible system, while *M. marinum* *ligD* and *nrgA* are expressed constitutively; Kanamycin resistance. | This study |
| pAK-Cpf1 | pNHEJ-Cpf1-derived plasmid; Cpf1 is expressed under a tetracycline-inducible system, while *M. marinum* *nrgA* and *ku* are expressed constitutively; Kanamycin resistance. | This study |
| **pNHEJ-Cpf1 (*ligD*^Mab^)** | pNHEJ-Cpf1-derived plasmid; Cpf1 is expressed under a tetracycline-inducible system, while *M. marinum* *nrgA* and *ku*, *M. abscessus ligD* are expressed constitutively; Kanamycin resistance. | This study |
| **pNHEJ-Cpf1 (*ku*^Mab^)** | pNHEJ-Cpf1-derived plasmid; Cpf1 is expressed under a tetracycline-inducible system, while *M. marinum* *nrgA* and *ligD*, *M. abscessus ku* are expressed constitutively; Kanamycin resistance. | This study |
| **pNHEJ-Cpf1 (NHEJ^Mab^+*nrgA*^Mmr^)** | pNHEJ-Cpf1-derived plasmid; Cpf1 is expressed under a tetracycline-inducible system, while *M. marinum* *nrgA* and *ligD*, *M. abscessus ku* and *ligD* are expressed constitutively; Kanamycin resistance. | This study |
| **pNHEJ-Cpf1 (NHEJ^Mab^)** | pNHEJ-Cpf1-derived plasmid; Cpf1 is expressed under a tetracycline-inducible system, while *M. abscessus ku* and *ligD* are expressed constitutively; Kanamycin resistance. | This study |
| pNHEJX-Cpf1(*recX*^Mab^) | pNHEJ-Cpf1-derived plasmid; Cpf1 is expressed under a tetracycline-inducible system, while *M. abscessus recX* is expressed constitutively; Kanamycin resistance. | This study |
| pNHEJX-Cpf1(*recX*^Msm^) | pNHEJ-Cpf1-derived plasmid; Cpf1 is expressed under a tetracycline-inducible system, while *M. smegmatis recX* is expressed constitutively; Kanamycin resistance. | This study |
| pNHEJ-SacB | pNHEJ-recX-sacB-derived plasmid; *M. marinum* NHEJ is expressed constitutively; Kanamycin resistance. | This study |
| pNHEJ-SacB(*recX*^Msm^) | pNHEJ-recX-sacB-derived plasmid; *M. smegmatis recX* is expressed constitutively, while *M. marinum* NHEJ is expressed constitutively; Kanamycin resistance. | This study |
| pCR-Zeo(carrying crRNA) | Used for expressing crRNA. Zeocin resistance. | This study |
| pZEO2085(carrying sgRNA) | Used for expressing sgRNA. Zeocin resistance. | This study |

**Table S2.** Genes knocked out in *M. abscessus* using the CRISPR-NHEJ strategy.

| Genes | Corresponding PAM and Protospacer Sequences | Possible Functions |
| --- | --- | --- |
| *MAB_0032* | **TTC**-TATGACCGCGGCGCCACCTTTA | Glutamine amidotransferase of anthranilate |
| *MAB_0490c* | **TTC**-GAGTCCGAAGGCTCCGAAGCGG | Putative adenylate cyclase |
| *MAB_0534* | **TTC**-GACGAGCAGCATGACGAGCTGG | GTP cyclohydrolase I (FolE) |
| *MAB_0535* | **TTC**-ATCCACACACCGGCTACCCG | Dihydropteroate synthase (FolP) |
| *MAB_0536* | **TTC**-GGTTCACCTTTGGCGCCGCGTT | Dihydroneopterin aldolase (FolB) |
| *MAB_0537* | **TTC**-CGATGACCCGGATCTCACGCTG | 2-amino-4-hydroxy-6- hydroxymethyldihydropteridine diphosphokinase (FolK) |
| *MAB_0540* | **TTC**-GCCGGTGGCGACGAGGATATC | Conserved hypothetical protein |
| *MAB_1055c* | **CTC**-GCGACACCCCCACCGACAAACT | Conserved hypothetical protein (peptidase?) |
| *MAB_1124c* | **TTC**-CGGCCGCGGCCGGCGGCTGGAC | Probable para-aminobenzoate synthase component I |
| *MAB_1345* | **TTC**-TATGACCGCGGCGCCACCTTTA | Probable dihydropteroate synthase 2 FolP2 |
| *MAB_1483* | **TTG**-GCCTCTGCGATGACATCCTCAC | Conserved hypothetical protein |
| *MAB_2217c* | **TTC**-AGGAGCCGCGCCTTTTTCCGTG | Sulfonate ABC transporter, ATP-binding subunit SsuB |
| *MAB_2245* | **TTC**-GGCACCTACCGATTCAATCTTCAG | Putative anthranilate synthase component I |
| *MAB_2297c* | **TTC**-ACGGTTTGCCGAGGAAGATGTC | Probable methyltransferase |
| *MAB_2362* | **TTC**-TCCGGGAACACCATCGAGTTCA | Conserved hypothetical protein |
| *MAB_2363* | **TTC**-CGGACTGCTCAACGACACGCTG | Conserved hypothetical protein |
| *MAB_2838c* | **TTC**-CGAGGTCCTGCTTCTTGAAAG | Putative cytoplasmic peptidase PepQ |
| *MAB_2976* | **TTC**-CGGCGAGCTGATCGACCTGTAT | Conserved hypothetical protein (possible reductase) |
| *MAB_3130c* | **TTC**-CGACGAGCCGGATTTCGCCGGC | Ribosome-binding factor A (RbfA) |
| *MAB_3513c* | **CTG**-GGTAGCCAGCCGTGGCCTTTC | Putative NADH pyrophosphatase/NUDIX hydrolase |
| *MAB_3534* | **TTC**-TCAGCAGCCCGCGCCGCCGCGC | Possible phosphoglycerate mutase EntD |
| *MAB_3535* | **TTG**-GTGGGTGCCCTGCCGTTCGACA | Probable isochorismate synthase EntC |
| *MAB_3837c* | **TTC**-GACTCTCACCTCGGGGTGATGC | Probable transcriptional regulatory proteinTetR |
| *MAB_3928c* | **TTC**-GATCTGATCGCAGCGCGAC | Probable polyprenyl-diphosphate synthase GrcC1 |
| *MAB_3938* | **CTG**-TCGTCGGTGCGGCAGGCTTTGCG | Putative Clp protease subunit |
| *MAB_3959c* | **TTC**-GAAACACTCTCGCGCACCTGA | Putative ATP-dependent Clp protease |
| *MAB_4059c* | **TTC**-GGTGGCCTCGCACGTGGACG | Conserved hypothetical protein |
| *MAB_4132* | **TTC**-TCGCCCTCCGAACTGGACATCC | Hypothetical protein |
| *MAB_4395* | **TTG**-CGGTGGATAGCGACTGGCGTGG | Aminoglycoside 2'-N-acetyltransferase |
| *MAB_4700c* | **TTG**-CGGTATTTGCACTGTCCCTGC | Conserved hypothetical protein |

**Table S3.** Primary primers used in this study (nucleotides **in bold** indicate genomic sequences, the remainder represent the recombinant sequences).

| Primers | Sequences (all 5’ to 3’) | Note |
| --- | --- | --- |
| MmNHEJ-F | GTAGTCTGCGCTTCAAAGCTT**ACAACACCCCGACACGCTCAC** | The forward primer amplifies the *M. marinum* NHEJ element for the construction of the pNHEJ-Cpf1 or pNHEJX-Cpf1 plasmids. |
| MmNHEJ-R | TGAGACACAA**GACTTCACGCAAAAACTCCTCTC** | The reverse primer amplifies the *M. marinum* NHEJ element for the construction of the pNHEJ-Cpf1 or pNHEJX-Cpf1 plasmids. |
| Kan-F | GCGTGAAGTC**TTGTGTCTCAAAATCTCTGATGTTACA** | The forward primer amplifies *kanR* fragment for the construction of the pNHEJ-Cpf1 or pNHEJX-Cpf1 plasmids. |
| Kan-R | ATTATACGAAGTTATGAATTC**TTGTAGGTGGACCAGTTGGTGA** | The reverse primer amplifies *kanR* fragment for the construction of the pNHEJ-Cpf1 or pNHEJX-Cpf1 plasmids. |
| HSP-F | CCGTGGCGCGGCCGCGGTACC**GGTGACCACAACGACGCG** | The forward primer amplifies *hsp* promoter for the construction of the pNHEJX-Cpf1 plasmid. |
| HSP-R(Mab-recX) | AGGACGTCAT**CGCAATTGTCTTGGCCATTG** | The reverse primer amplifies *hsp* promoter for the construction of the pNHEJX-Cpf1(*recX*^Mab^) plasmid. |
| HSP-R(Mab-recA) | TGCGCCAT**CGCAATTGTCTTGGCCATTG** | The reverse primer amplifies *hsp* promoter for the construction of the pNHEJA-Cpf1 plasmid. |
| HSP-R(Mab-recO) | AAAGCCGCAT**CGCAATTGTCTTGGCCATTG** | The reverse primer amplifies *hsp* promoter for the construction of the pNHEJO-Cpf1 plasmid. |
| HSP-R(Msm-recX) | CGACTTCGTCAT**CGCAATTGTCTTGGCCATTG** | The reverse primer amplifies *hsp* promoter for the construction of the pNHEJX-Cpf1(*recX*^Msm^) plasmid. |
| Mab-recX-F | CAATTGCG**ATGACGAAGTCGTCCCGGC** | The forward primer amplifies *recX*^Mab^ for the construction of the pNHEJX-Cpf1(*recX*^Mab^) plasmid. |
| Mab-recX-R | TAGATTTAAAGATCTGGTACC**TCATCCGACGTCGCGTCG** | The reverse primer amplifies *recX*^Mab^ for the construction of the pNHEJX-Cpf1(*recX*^Mab^) plasmid. |
| Mab-recA-F | AAGACAATTGCG**ATGGCGCAGGCACCGGAT** | The forward primer amplifies *recA*^Mab^ for the construction of the pNHEJA-Cpf1 plasmid. |
| Mab-recA-R | TAGATTTAAAGATCTGGTACC**GGTTCACGCGTCGGGGCT** | The reverse primer amplifies *recA*^Mab^ for the construction of the pNHEJA-Cpf1 plasmid. |
| Mab-recO-F | GACAATTGCG**ATGCGGCTTTATCGGGATCG** | The forward primer amplifies *recO*^Mab^ for the construction of the pNHEJO-Cpf1 plasmid. |
| Mab-recO-R | TAGATTTAAAGATCTGGTACC**GGGAAAACAGCAGGAAACACC** | The reverse primer amplifies *recO*^Mab^ for the construction of the pNHEJO-Cpf1 plasmid. |
| Msm-recX-F | GACAATTGCG**ATGACGTCCTCCCGGCCC** | The forward primer amplifies *recX*^Msm^ for the construction of the pNHEJX-Cpf1(*recX*^Msm^) plasmid. |
| Msm-recX-R | TAGATTTAAAGATCTGGTACC**CTAGACCCGCCGGCGCTC** | The reverse primer amplifies *recX*^Msm^ for the construction of the pNHEJX-Cpf1(*recX*^Msm^) plasmid. |
| MmNHEJ-F(SacB) | GGTGGCATCCGTGGCGCGGCCGC**ACAACACCCCGACACGCTC** | The forward primer amplifies the *M. marinum* NHEJ element for the construction of the pNHEJ-SacB plasmids. |
| MmNHEJ-R(SacB) | CGGCGGCACGACGAGCATATG**TGAACAAGTCGGGCGCTG** | The reverse primer amplifies the *M. marinum* NHEJ element for the construction of the pNHEJ-SacB or pNHEJX-SacB(*recX*^Msm^) plasmids. |
| MmNHEJ-F2(SacB) | TCAAAGCTT**ACAACACCCCGACACGCTC** | The forward primer amplifies the *M. marinum* NHEJ element for the construction of the pNHEJX-SacB(*recX*^Msm^) plasmids. |
| RecX-F(SacB) | GGTGGCATCCGTGGCGCGGCCGC**GGTGACCACAACGACGCG** | The forward primer amplifies *recX*^Msm^ with *hsp* promoter for the construction of the pNHEJX-SacB(*recX*^Msm^) plasmid. |
| RecX-R(SacB) | CGGGGTGTTGT**AAGCTTTGAAGCGCAGACTACAC** | The reverse primer amplifies *recX*^Msm^ with *hsp* promoter for the construction of the pNHEJX-SacB(*recX*^Msm^) plasmid. |
| MAB_3513c-ci-F | AT**GGTAGCCAGCCGTGGCCTTTC**A | Forward primer for constructing crRNA targeting the *MAB_3513c* gene. |
| MAB_3513c-ci-R | AGCTT**GAAAGGCCACGGCTGGCTACC**ATCT | Reverse primer for constructing crRNA targeting the *MAB_3513c* gene. |
| MAB_3513-F | **ATGACCTTCCGCCTTCGC** | Forward primer for amplifying *MAB_3513c* gene. |
| MAB_3513-R | **CTACGGTCTTCCCGCAGGC** | Reverse primer for amplifying *MAB_3513c* gene. |
| MSM_1946-ci-F | AT**GAGACATGCGTGCAGCGCGAG**A | Forward primer for constructing crRNA targeting the *MSMEG_1946* gene. |
| MSM_1946-ci-R | AGCTT**CTCGCGCTGCACGCATGTCTC**ATCT | Reverse primer for constructing crRNA targeting the *MSMEG_1946* gene. |
| MSM_1946-F | **TCCGAGCGTGGTGGAGATGA** | Forward primer for amplifying *MSMEG_1946* gene. |
| MSM_1946-R | **CCGTACCCGCAGGCGTAATT** | Reverse primer for amplifying *MSMEG_1946* gene. |
| MAB_0490c-ci-F | AT**GAGTCCGAAGGCTCCGAAGCGG**A | Forward primer for constructing crRNA targeting the *MAB_0490c* gene. |
| MAB_0490c-ci-R | AGCTT**CCGCTTCGGAGCCTTCGGACTC**ATCT | Reverse primer for constructing crRNA targeting the *MAB_0490c* gene. |
| MAB_0534-ci-F | AT**GACGAGCAGCATGACGAGCTGG**A | Forward primer for constructing crRNA targeting the *MAB_0534* gene. |
| MAB_0534-ci-R | AGCTT**CCAGCTCGTCATGCTGCTCGTC**ATCT | Reverse primer for constructing crRNA targeting the *MAB_0534* gene. |
| MAB_0535-ci-F | AT**ATCCACACACCGGCTACCCG**A | Forward primer for constructing crRNA targeting the *MAB_0535* gene. |
| MAB_0535-ci-R | AGCTT**CGGGTAGCCGGTGTGTGGAT**ATCT | Reverse primer for constructing crRNA targeting the *MAB_0535* gene. |
| MAB_0537-ci-F | AT**CGATGACCCGGATCTCACGCTG**A | Forward primer for constructing crRNA targeting the *MAB_0537* gene. |
| MAB_0537-ci-R | AGCTT**CAGCGTGAGATCCGGGTCATCG**ATCT | Reverse primer for constructing crRNA targeting the *MAB_0537* gene. |
| MAB_1345-ci-F | AT**TATGACCGCGGCGCCACCTTTA**A | Forward primer for constructing crRNA targeting the *MAB_1345* gene. |
| MAB_1345-ci-R | AGCTT**TAAAGGTGGCGCCGCGGTCATA**ATCT | Reverse primer for constructing crRNA targeting the *MAB_1345* gene. |
| MAB_2217c-ci-F | AT**AGGAGCCGCGCCTTTTTCCGTG**A | Forward primer for constructing crRNA targeting the *MAB_2217c* gene. |
| MAB_2217c-ci-R | AGCTT**CACGGAAAAAGGCGCGGCTCCT**ATCT | Reverse primer for constructing crRNA targeting the *MAB_2217c* gene. |
| MAB_2297c-ci-F | AT**ACGGTTTGCCGAGGAAGATGTC**A | Forward primer for constructing crRNA targeting the *MAB_2297c* gene. |
| MAB_2297c-ci-R | AGCTT**GACATCTTCCTCGGCAAACCGT**ATCT | Reverse primer for constructing crRNA targeting the *MAB_2297c* gene. |
| MAB_2362c-ci-F | AT**TCCGGGAACACCATCGAGTTCA** | Forward primer for constructing crRNA targeting the *MAB_2362* gene. |
| MAB_2362c-ci-R | AGCTT**TGAACTCGATGGTGTTCCCGGA** | Reverse primer for constructing crRNA targeting the *MAB_2362* gene. |
| MAB_3130c-ci-F | AT**CGACGAGCCGGATTTCGCCGGC**A | Forward primer for constructing crRNA targeting the *MAB_3130c* gene. |
| MAB_3130c-ci-R | AGCTT**GCCGGCGAAATCCGGCTCGTCG**ATCT | Reverse primer for constructing crRNA targeting the *MAB_3130c* gene. |
| MAB_4059c-ci-F | AT**GGTGGCCTCGCACGTGGACG**A | Forward primer for constructing crRNA targeting the *MAB_4059c* gene. |
| MAB_4059c-ci-R | AGCTT**CGTCCACGTGCGAGGCCACC**ATCT | Reverse primer for constructing crRNA targeting the *MAB_4059c* gene. |
| MAB_4132-ci-F | AT**TCGCCCTCCGAACTGGACATCC**A | Forward primer for constructing crRNA targeting the *MAB_4132* gene. |
| MAB_4132-ci-R | AGCTT**GGATGTCCAGTTCGGAGGGCGA**ATCT | Reverse primer for constructing crRNA targeting the *MAB_4132* gene. |
| MAB_4395-ci-F | AT**CGGTGGATAGCGACTGGCGTGG**A | Forward primer for constructing crRNA targeting the *MAB_4395* gene. |
| MAB_4395-ci-R | AGCTT**CCACGCCAGTCGCTATCCACCG**ATCT | Reverse primer for constructing crRNA targeting the *MAB_4395* gene. |
| MAB_0490c-F | **GTGACTGGCGCGGAGGGT** | Forward primer for amplifying *MAB_0490c* gene. |
| MAB_0490c-R | **TTAGCCGTGACGCGGGCG** | Reverse primer for amplifying *MAB_0490c* gene. |
| MAB_0534-F | **ATGAACTCGCCCGAGACG** | Forward primer for amplifying *MAB_0534* gene. |
| MAB_0534-R | **TCATGTCCTCATCATCAAGTCAAGAACCTCGG** | Reverse primer for amplifying *MAB_0534* gene. |
| MAB_0535-F | **ATGATCGCTGGCGTGACG** | Forward primer for amplifying *MAB_0535* gene. |
| MAB_0535-R | **TCATTTCGGTTCACCTTTGGCGCC** | Reverse primer for amplifying *MAB_0535* gene. |
| MAB_0537-F | **GTGCGTGCCGTGCTTTCGA** | Forward primer for amplifying *MAB_0537* gene. |
| MAB_0537-R | **CTACGTCACCGGACCGG** | Reverse primer for amplifying *MAB_0537* gene. |
| MAB_1345-F | **ATGGCGAGGCGGGCTG** | Forward primer for amplifying *MAB_1345* gene. |
| MAB_1345-R | **TCATGCCAACCCCCTCACTGTTCGC** | Reverse primer for amplifying *MAB_1345* gene. |
| MAB_2217c-F | **ATGACAGCGGTCTTCGAGGTC** | Forward primer for amplifying *MAB_2217* gene. |
| MAB_2217c-R | **AAACACCCAACTGTGCAAGG** | Reverse primer for amplifying *MAB_2217* gene. |
| MAB_2297c-F | **GTGTCCGGCCAACGGTCG** | Forward primer for amplifying *MAB_2297c* gene. |
| MAB_2297c-R | **CAGCGCCGCCTGATCAC** | Reverse primer for amplifying *MAB_2297c* gene. |
| MAB_2362-F | **ATGATCACCCCTATGAACTTGAC** | Forward primer for amplifying *MAB_2362* gene. |
| MAB_2362-R | **TCAGCTGACCAGGTTCTGCAC** | Reverse primer for amplifying *MAB_2362* gene. |
| MAB_3130c-F | **GTGGCAGATCCCGCCCGC** | Forward primer for amplifying *MAB_3130c* gene. |
| MAB_3130c-R | **TCAATCGGGACGGCGCTC** | Reverse primer for amplifying *MAB_3130c* gene. |
| MAB_4059c-F | **ATGACCGCACCAGTTCGCC** | Forward primer for amplifying *MAB_4059c* gene. |
| MAB_4059c-R | **CTAAGCCAGCGCGGAGGC** | Reverse primer for amplifying *MAB_4059c* gene. |
| MAB_4132-F | **ATGACAAACAACCTATTCGTCGG** | Forward primer for amplifying *MAB_4132* gene. |
| MAB_4132-R | **CTAGGCGGCGTGAGCGTC** | Reverse primer for amplifying *MAB_4132* gene. |
| MAB_4395-F | **ATGTCGGCTGTGTCCAATATGC** | Forward primer for amplifying *MAB_4395* gene. |
| MAB_4395-R | **TCACCAGCCGTCGCCGGC** | Reverse primer for amplifying *MAB_4395* gene. |

**Reference**

1. Wang S, Zhang J, Hameed HMA, Ding J, Guan P, Fang X, et al. Amino acid 17 in QRDR of Gyrase A plays a key role in fluoroquinolones susceptibility in mycobacteria. Microbiol Spectr. 2023;11(6):e0280923.
